# Impact of Adverse Mobility Ratio on Oil Mobilization by Polymer Flooding

**DOI:** 10.3390/polym17152033

**Published:** 2025-07-25

**Authors:** Abdulmajeed Murad, Arne Skauge, Behruz Shaker Shiran, Tormod Skauge, Alexandra Klimenko, Enric Santanach-Carreras, Stephane Jouenne

**Affiliations:** 1 Department of Chemistry, University of Bergen, 5007 Bergen, Norway; 2 Institute of GeoEnergy Engineering, Heriot-Watt University, Edinburgh EH14 4AS, UK; 3 Energy Research Norway, 5147 Fyllingsdalen, Norway; tormod.skauge@energyresearch.no; 4 NORCE Norwegian Research Centre AS, 5008 Bergen, Norway; besh@norceresearch.no; 5 TotalEnergies SA, Pôle d’Etudes et Recherche de Lacq, BP 47, 64170 Lacq, France; alexandra.klimenko@totalenergies.com (A.K.); enric.santanach-carreras@totalenergies.com (E.S.-C.); stephane.jouenne@totalenergies.com (S.J.)

**Keywords:** polymer flooding, enhanced oil recovery, fluid flow, viscous oil, adverse mobility ratio, energy efficiency

## Abstract

Polymer flooding is a widely used enhanced oil recovery (EOR) method for improving energy efficiency and reducing the carbon footprint of oil production. Optimizing polymer concentration is critical for maximizing recovery while minimizing economic and environmental costs. Here, we present a systematic experimental study which shows that even very low concentrations of polymers yield relatively high recovery rates at adverse mobility ratios (230 cP oil). A series of core flood experiments were conducted on Bentheimer sandstone rock, with polymer concentrations ranging from 40 ppm (1.35 cP) to 600 ppm (10.0 cP). Beyond a mobility ratio threshold, increasing polymer concentration did not significantly enhance recovery. This plateau in performance was attributed to the persistence of viscous fingering and oil crossflow into pre-established water channels. The study suggests that low concentrations of polymer may mobilize oil at high mobility ratios by making use of the pre-established water channels as transport paths for the oil and that the rheology of the polymer enhances this effect. These findings enable reductions in the polymer concentration in fields with adverse mobility ratios, leading to substantial reductions in chemical usage, energy consumption, and environmental impact of the extraction process.

## 1. Introduction

Oil recovery is most efficient when it is supported by injected water or gas. This allows the reservoir’s pressure to be maintained and for the injected phase to displace the oil. The purpose of adding polymers to the injected water is to increase its viscosity. An oil reservoir is heterogeneous with regard to flow permeability and may have barriers such as sealing fractures and impermeable shale or clay layers. Increasing the viscosity of the injected water reduces its mobility in the areas where it is preferentially flowing, diverting the flow to areas not previously contacted. This is the main principle behind polymer injection: to improve areal sweep.

Polymer flooding has received considerable attention ever since its first commercial application in the 1960s [1,2,3,4,5]. The benefits of introducing polymer flooding strategically in a field development plan include the acceleration of oil production and the reduction of water-cut and water treatment. Reducing the energy invested for energy return is a critical aspect of moving towards a net-zero society. Recent studies report that polymer flooding may reduce the overall CO_2_ emissions by as much as 35–50% compared to flooding by water [6].

During conventional water flooding in heavy oil, the sharp viscosity contrast between heavy oil and water creates unstable displacement. Injected water fingers through the oil and leaves significant amounts of bypassed oil in the reservoir [7,8]. This phenomenon is known as viscous fingering, and it has negative effects like early water breakthroughs, high water-cut, and low oil recovery. In heterogeneous reservoirs, water finds the least resistant path through high-permeability regions, bypassing the oil in the low-permeability regions. In these scenarios, the mobility ratio (Equation (1)) is greater than unity. The mobility of a phase is governed by its viscosity and effective permeability. The ratio of displacing fluid to displaced fluid mobility is the mobility ratio (*M*) [9], and for water flooding it is defined as

(1) $$M=\frac{\lambda_{w}}{\lambda_{o}}=\frac{\left(\frac{k_{rw}}{\mu_{w}}\right)}{\left(\frac{k_{ro}}{\mu_{o}}\right)}=\frac{k_{rw}}{k_{ro}}\cdot \frac{\mu_{o}}{\mu_{w}}$$

where λ is the mobility, *k_r_* is the relative permeability, *µ* is the viscosity, and the subscripts ‘w’ and ‘o’ denote water and oil, respectively.

Traditional screening criteria published up to the millennium concluded that technical and economic factors restricted the practical application of polymer flooding in heavy oil reservoirs [3,4,5,10,11]. The recommended target for polymer flooding was a mobility ratio of less than one (M < 1), which required prohibitively high polymer concentrations for viscous oils. Thus, polymer flooding was not recommended for oil viscosity greater than 150 cP. Then, in 2006, polymer flooding was applied in a field pilot at the Pelican Lake reservoir in Canada [12]. In the pilot region, the oil viscosity was between 1000 and 1500 cP, far above the previous limits of polymer flooding applicability. By injecting only 20 cP polymer solution, far from offering unit mobility, the oil recovery was increased to 15–30% of original oil in place (OOIP) compared to the increase of around 10%OOIP achieved by water flooding. Part of this increase in efficiency was credited to horizontal wells. Horizontal wells have much longer sections, open to injection and production in the reservoir layer, and therefore increase areal sweep compared to vertical wells. However, it was difficult to explain why the injection of relatively low polymer concentrations could work so well in terms of mobilizing heavy oil, even with horizontal wells.

Around the same time, but unrelated, laboratory studies were started in Bergen, Norway [7,8]. They used square slabs of outcrop rock for oil displacement experiments involving water and polymers. These experiments were mimicking the injector–producer pairs of horizontal wells, different from the traditional core flood studies that use 1–2-inch cylindrical cores. Monitoring the oil production by x-ray imaging, the viscous fingering pattern of unstable displacement was clearly seen. Moreover, the oil production profiles seemed similar to those observed in the field in Canada [12,13,14,15].

The original screening criteria for polymers assumed that viscous fingering did not influence the displacement significantly. X-ray images from the lab showed that the polymer mobilized oil into the established water channels, where the oil was pushed to the producer and recovered [8,16,17]. In this scenario, only a small increase in injected fluid viscosity was necessary to mobilize oil into the water channels. This process, known as viscous crossflow [18], could explain why a low concentration of polymer could lead to rapid oil production and high sweep efficiency. There is experimental evidence for oil mobilization at low polymer concentrations in slab and core experiments [19]. A methodology for simulating viscous crossflow has been developed based on the data, and it has produced a good match of the flow behavior up to water breakthrough [20,21,22,23]. New screening criteria were necessary to accommodate the crossflow mechanism in line with the field observations. Recently, the maximum oil viscosity limit for polymer application was reported as >10,000 cP [24]. Currently, heavy oil is produced by polymer flooding at Milne Point in Alaska [25,26,27], and at Pelican Lake [28] and East Bodo [29] in Canada. In addition, recent pilots were reported in Oman [30], India [31] and Argentina [32].

After the success at Pelican Lake, heavy oil fields could now achieve energetic, environmental and economic gain by injecting polymer. However, the question of how much polymer was required to mobilize oil at adverse mobility remained. In Canada, Wang, and Dong [33,34] tried to answer this by examining the effective viscosity of polymer required to improve the recovery of heavy oil in sand-pack columns. They found that tertiary oil recovery as a function of effective viscosity exhibited an S-shaped curvature. It seemed that there was a minimum polymer viscosity threshold for mobilizing oil (i.e., the lower plateau), and a maximum viscosity (i.e., upper plateau) where a further increase resulted in little to no effect. For an oil viscosity of 430 cP, the minimum polymer effective viscosity was 9 cP, and for 2000 cP oil it was 30 cP. Regarding cylindrical outcrop cores and square slabs, [19,35] showed that 3 cP polymer significantly increased the recovery of 2000 cP oil, a concentration 10 times lower than that reported in [33,34]. A key difference is that [33,34] used unconsolidated sand as porous medium, whereas [19,35] used consolidated sandstone material.

This study was initiated to further investigate the limits of polymer concentration for oil mobilization at adverse mobility in consolidated rock. Here, we present a systematic study of oil mobilization and displacement characteristics for water floods at adverse mobility, followed by tertiary polymer floods. The objectives of this study are to quantitatively describe oil mobilization by polymers in consolidated sandstone material, and to improve our understanding of why low-concentration polymer may be so effective in heavy oil mobilization. In addition, we investigate the effect of wettability and the effect of secondary (polymer displacing oil) and tertiary (polymer displacement after waterflood) displacement on recovery efficiency.

## 2. Materials and Methods

### 2.1. Fluids

Synthetic brine, mineral oil, and heavy Stock Tank Oil (STO) were used. The synthetic brine composition was 0.6 wt% NaCl and 0.1 wt% NaHCO_3_ and its preparation involved the dissolution of lab-grade salts in deionized water followed by filtration, through a 0.45 µm filter. It was used for core saturation, polymer solution preparation, and water flooding. Its viscosity was 1.02 cP at 22 °C. The heavy crude oil was used for the drainage and aging processes. The crude oil was filtered through a 0.5 µm filter before use and its viscosity was 230 cP at 22 °C. Primol 542 mineral oil was used as a non-wetting oil, and its viscosity was 250 cP at 22 °C.

### 2.2. Porous Medium

The physical properties of the core samples are listed in Table 1. Outcrop Bentheimer sandstone was used as the porous medium. The core samples were cut and then dried in a heating cabinet overnight at 100 °C. The cores were then mounted in a core holder, vacuumed, and saturated with synthetic brine. After that, the cores were drained vertically by the injection of oil from the top. The injection rate started at 0.05 mL/min and then increased to 0.1 and 0.3 mL/min until no more water was produced. The injection rate was then reduced stepwise until the differential pressure stabilized and the effective oil permeability before aging ($k_{o}(S_{wirr}$)) was calculated. Irreducible water saturation ($S_{wirr}$) and the original oil in place (OOIP) were calculated from mass balance, where OOIP = ${1-S}_{wirr}$. The cores were aged at 50 °C with 10 bar back-pressure for a month while injecting crude oil at a rate of 1 pore volume (PV)/week. The effective oil permeability after aging was measured again at ambient conditions. The sample BEN10 was drained by mineral oil only to maintain a water-wet state and subsequently was not aged. The core flooding experiments were performed at a constant flow rate of 0.1 mL/min, which corresponded to a Darcy velocity of 0.4 ft/d (12.2 cm/day), representative of expected frontal velocity in the reservoir.

### 2.3. Polymer

**Figure 1:**
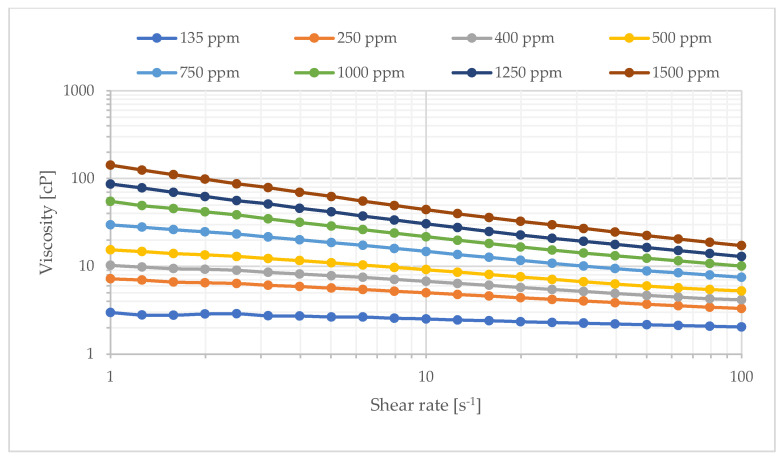
Shear viscosity of select concentrations of FP3630s at 22 °C.

The partially hydrolyzed polyacrylamide (HPAM) Flopaam (FP) 3630s with a molecular weight of approximately 18 × 10^6^ g/mol was used to prepare polymer solutions. It was supplied in powder form with an activity of 90%, delivered from SNF SA, Andreziuex, France. Initially, a stock solution of 5000 ppm concentration was prepared and mixed overnight at a low speed. It was diluted with brine to prepare polymer solutions of various concentrations. In total, 5 polymer concentrations were selected. The polymer solutions were filtered through 5 µm filter before viscosity measurements and injection. Stock solution preparation was determined according to the API procedure RP-63 [36]. The bulk viscosity of the polymer solutions as a function of shear rate was measured by the cone-plate geometry in a rotational viscometer at 22 °C. Bulk viscosities are reported at a shear rate of 10 s^−1^ unless specifically noted. Shear rheology curves for select concentrations are shown in Figure 1.

### 2.4. Experimental Setup

**Figure 2:**
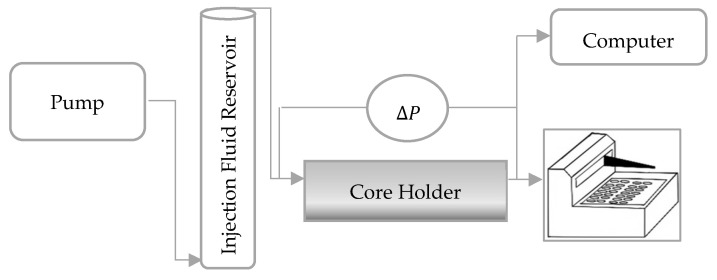
Schematic of core flooding apparatus (∆P: differential pressure).

The core flooding apparatus consisted of a Quizix precision pump, a floating-piston cylinder, a core holder, and a fraction collector. The floating-piston cylinder functioned as the injection fluid reservoir and was operated by the pump. The differential pressure across the core was continuously measured by a FUJI pressure transduce and recorded by a computer. Figure 2 illustrates the experimental setup.

## 3. Results and Discussion

All cores, except for BEN09, were first flooded by brine (water flood), followed by polymer solution (polymer flood), and again by brine. BEN09, on the other hand, was flooded with polymer solution without a proceeding water flood. The injection rate was 0.1 mL/min, unless stated otherwise, and the temperature was 22 °C. The injection volume was decided in advance as approximately 4 PV. Though it would be excessive for field application, it ensured oil production was complete, and differential pressure was stable for all experiments.

### 3.1. Effect of Polymer Solution Viscosity

The viscosity of polymer solution was varied to study its effect on oil recovery. Table 2 summarizes the main results of 5 of the core flooding experiments.

**Figure 3:**
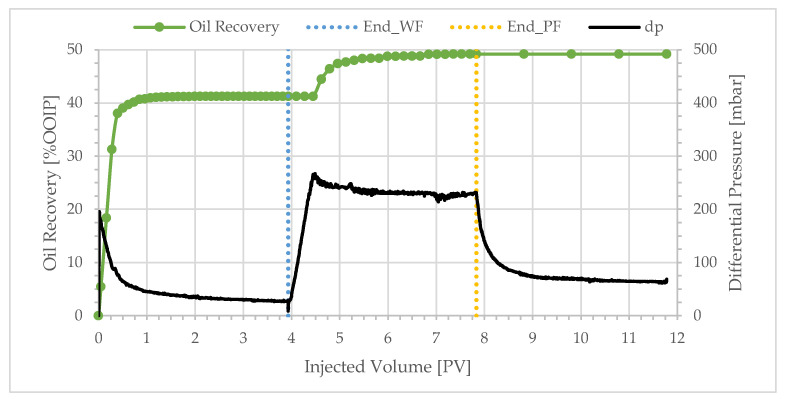
Oil recovery and differential pressure profiles of BEN05 (polymer viscosity: 10.0 cP).

The bulk viscosity of the injected polymer solution ranged from 10.0 to 1.35 cP. The average oil recovery after the water flooding of these specific experiments was 40.3% and the incremental recovery after polymer flooding averaged 7.2%. The unexpected result in this series of experiments was the high extra oil production achieved at low polymer viscosity. Figure 3 shows the production profile and differential pressure behavior for a polymer viscosity of 10.0 cP.

Over 90% of the oil production occurred during the injection of the first 0.5 PV, and the water-cut after the injection of only 1 PV was over 98%. The oil recovery after water flooding was low at only 41.23%, and the water breakthrough was early (WBT < 0.3 PV). The differential pressure rapidly peaked at 190 mbar—because of the high viscosity of the crude oil—and then gradually decreased and stabilized at approximately 27 mbar. The oil–water viscosity ratio was 225. The production profile, differential pressure behavior, low oil recovery, and early water breakthrough indicate unstable displacement. The instability of the flooding front was a result of significant viscous fingering—as expected at such an adverse mobility ratio. After the injection of 3.9 PV, the water flood was completed, and the injection fluid was switched to a polymer solution with a bulk viscosity of 10.0 cP at 10 s^−1^. Significant incremental oil production was not observed until 0.7 PV of the polymer solution was injected, coinciding with the differential pressure peak of 260 mbar. The late start of oil mobilization could be explained by polymer retention. The sharp increase in oil production that followed could be explained by crossflow—polymer driving the oil into water channels that were established during the proceeding water flood. The crossflow mechanism was derived from X-ray images of similar flood processes [8,18]. Oil production ceased after the next 1.6 PV; however, the injection of polymer solution continued until differential pressure was stable. At the end of the polymer flood, the total oil recovery was 49.2%, out of which 8.0% was achieved by polymer flooding. Oil recovery was insignificant during chase brine injection after the polymer flood.

**Figure 4:**
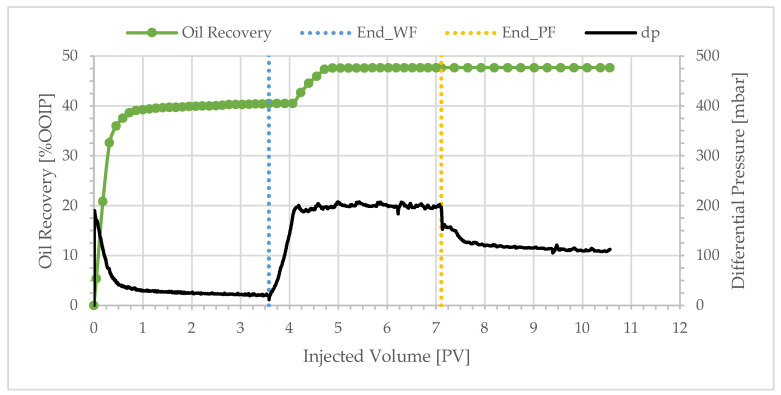
Oil recovery and differential pressure profiles of BEN04 (polymer viscosity: 4.1 cP).

**Figure 5:**
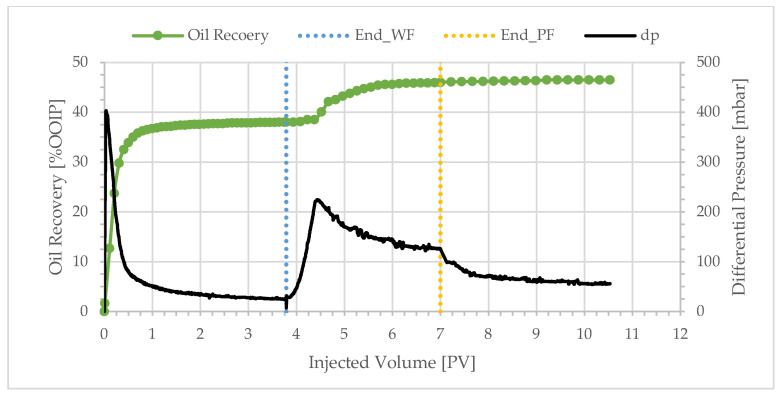
Oil recovery and differential pressure profiles of BEN03 (polymer viscosity: 2.53 cP).

**Figure 6:**
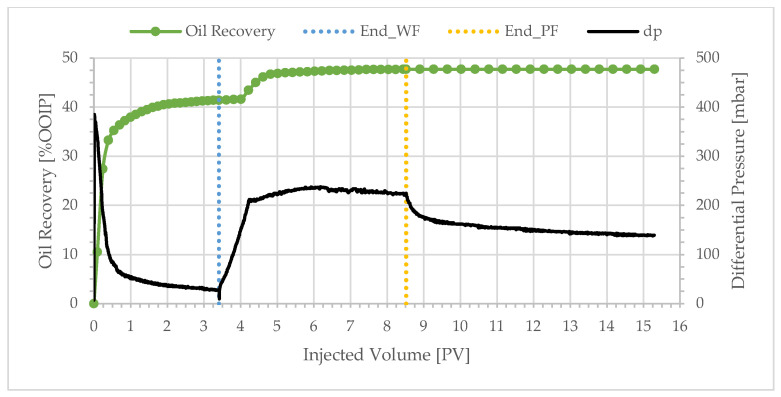
Oil recovery and differential pressure profiles of BEN06 (polymer viscosity: 1.8 cP).

**Figure 7:**
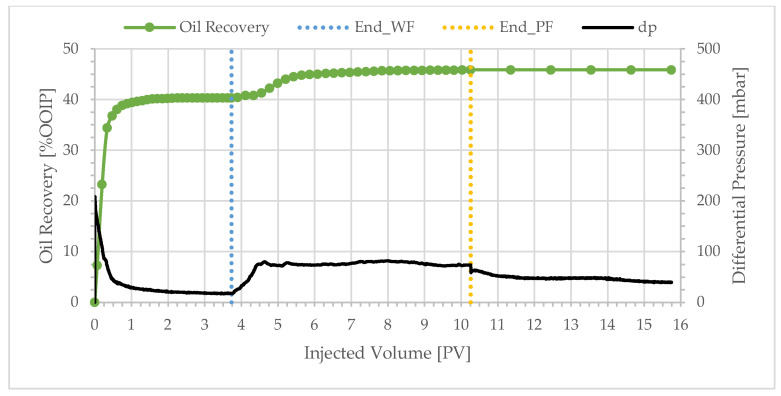
Oil recovery and differential pressure profiles of BEN08 (polymer viscosity: 1.35 cP).

Similar overall trends were observed with BEN04, BEN03, BEN06, and BEN08 as shown in Figure 4, Figure 5, Figure 6 and Figure 7. Oil production was fast and the total recovery after water flooding was below 50%. Water breakthrough was observed after injecting 0.2–0.3 PV. The differential pressure peaked rapidly and then decreased to a stable value of approximately 20 mbar. The subsequent injection of a polymer solution resulted in a delayed incremental recovery, and the total oil recovery remained unchanged during chase brine injection. Generally, the differential pressure during polymer injection stabilized close to the peak value.

**Figure 8:**
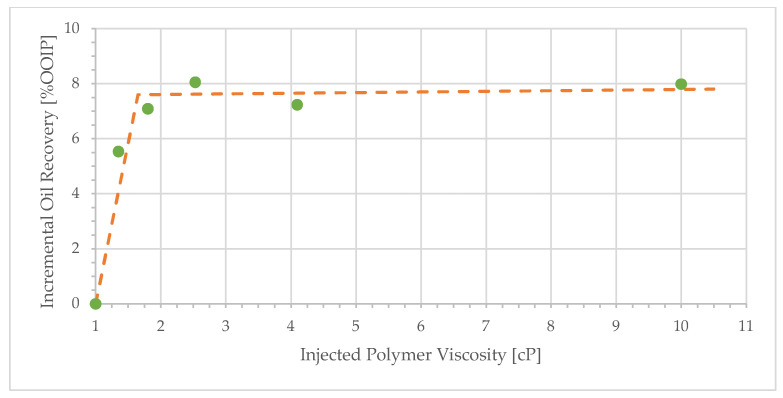
Incremental oil recovery as a function of injected solution bulk viscosity.

Decreasing the polymer solution bulk viscosity does not significantly affect the incremental oil recovery, as illustrated in Figure 8. For example, reducing the polymer solution viscosity from 10.0 to 1.8 cP (Figure 3 and Figure 6)—a factor of approximately 5.6—results in incremental recoveries of 8.1% and 7.1%, respectively. This reduction in bulk viscosity corresponds to a reduction in polymer concentration from 600 to 70 ppm—a factor of 9. The threshold value for the plateau in Figure 8 corresponds to a polymer concentration of approximately 50 ppm. Yet, even a polymer concentration of only 40 ppm leads to significant incremental oil recovery. It is evident that one can achieve significant incremental oil recovery with lower polymer concentration. These results indicate an economically attractive proposition: the injection of low polymer concentrations into heavy oil reservoirs in the presence (or absence) of water fingers. Experiments with a 209 cP oil were performed in high-permeability sandpacks [37]. The results were comparable to those reported here: a low-viscosity polymer solution of 4.0 cP achieved 71% oil recovery, only 6.5% lower than the recovery for the highest viscosity solution of 25.0 cP.

Polymer concentration as low as 70 ppm (1.8 cP) gives an incremental oil recovery of 7.1%. The viscosity ratio, o/p, for this displacement is 128, which is strongly unstable. Furthermore, the polymer flood performs similar to that of 10.0 cP. Both are far from the mobility ratio of 1, which is traditionally viewed as a requirement to achieve stable displacement. It may be partly explained by the flow resistance caused by the polymer.

The flow resistance is quantified by the resistance factor (RF), which is the relative differential pressure during the polymer flood, compared with that of the proceeding water flood, i.e., *RF =* Δ*P_p_*/Δ*P_w,prior_*. Several factors contribute to the differential pressure and often Darcy’s law is applied to separate the individual contributions:

(2) $$∆P=\frac{q\mu L}{AK}$$

where *q* is the flow rate, *µ* is the viscosity of the fluid, *L* is the length of the core, *A* is the cross-sectional area of the core, and *K* is the permeability. One interpretation of this relation is to assume that all the core related properties, *q*, *L*, *A* and *K,* are the same for water (w) and polymer (p) so that

(3) $$RF=\frac{∆P_{p}}{∆P_{w,prior}}=\frac{q_{p}\mu_{p}L_{p}}{A_{p}K_{p}}\cdot \frac{A_{w,prior}K_{w,prior}}{q_{w,prior}\mu_{w,prior}L_{w,prior}}≅\frac{\mu_{p}}{\mu_{w,\;prior}}=\mu_{rel}$$

where *µ_rel_* is the relative viscosity. Viscosities of polymer and water from bulk rheology measurements suggest RF values of 1.35–10 inasmuch as the water viscosity is approximately 1. But the calculated RF from differential pressure ranges from 4.1 to 9.5, as presented in Table 3.

Polymer will reduce the permeability of the porous media by adsorption, straining, and mechanical entrapment. This is characterized by the residual resistance factor:

(4) $$RRF=\frac{K_{w,\;prior}}{K_{w,\;after}}=\frac{dp_{w,after}}{dp_{w,prior}}$$

This decrease in permeability leads to greater differential pressure during the polymer flood compared with the water flood and therefore an increase in RF (Equation (3)). A requirement for RRF measurements is that fluid saturations remain constant before and after the polymer flood, which is not the case here. Relative permeability is a function of saturation, and therefore RF and RRF cannot be determined without knowing the relative permeability curve. Determining relative permeability under adverse mobility is a challenging task and beyond the scope of this paper. Nevertheless, RRF can be estimated from similar experiments and based on experience; RRF is expected to be in the order of 1.2–1.8 for relatively high-permeability Bentheimer rock with the presence of polymer and brine salinity. Similarly, an estimate of relative permeability at adverse mobility with an average saturation change from 0.47 to 0.54 suggests that *k_rp_*(*S_orp_*) ≈ 1.5 × *k_rw_*(*S_orw_*). Polymer retention (RRF) would increase polymer differential pressure, whereas the change in *k_r_* would reduce differential pressure, and in this case the two effects approximately cancel each other. Working from Equation (3), this can be written as

(5) $$K_{w,prior\;}=K_{abs}{\cdot k}_{rw}(S_{orw})\approx K_{p}=\frac{K_{abs}{\cdot k}_{rp}(S_{orp})}{RRF}$$

where *K_abs_* is the absolute permeability, *k_rw_*(*S_orw_*) is the relative permeability of water at remaining oil saturation after water flooding, *k_rp_*(*S_orp_*) is the relative permeability of polymer at remaining oil saturation after polymer flooding and the residual resistance factor, RRF, is defined in Equation (4).

Given that the injection rate, q, is equal for water and polymer, the only remaining factor in the RF relation, Equation (3), is the ratio *L*/*A*. While the core length and cross-sectional area do not change during the flooding, the effective area accessible to the injected fluid and the actual length of the flow path within the core will change from the water to the polymer flood. The flow path in the porous media is a function of the pore size distribution of rock, the pore throat to pore body aspect ratio, and the overall tortuosity of the system. These factors generate a contraction–expansion flow that, because of the viscoelastic nature of polymer, may induce an increase in the viscosity [38]. The magnitude of the increase in viscosity is strongly dependent on the pore-scale flow path.

**Figure 9:**
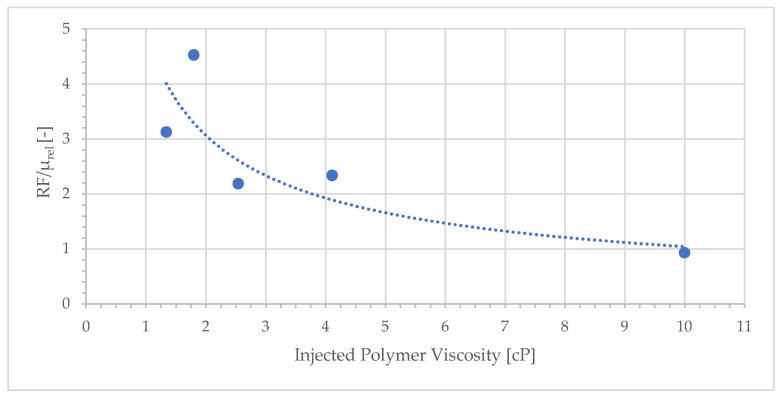
Relative pressure of polymer to water of core flooding (RF) normalized by relative bulk viscosity of polymer to water (µ_rel_) plotted as function of injected viscosity.

The application of Darcy’s law to core flood experiments assumes a capillary bundle model (CBM), in which flow in the porous media is represented by linear flow through a bundle of straight tubes with a given diameter [3]. Because polymer viscosity is a function of the local flow velocity, and consequently the dynamically changing pore size, the CBM is no longer valid. The increase in polymer viscosity is often referred to as shear thickening or rather, elastic turbulence [38], and leads to increased measured differential pressure. FP3630s is a large (18 MD) HPAM polymer and shows strong elastic turbulence. In contrast, measurements of shear viscosity, which are the industry standard, do not detect elastic turbulence effects. Consequently, and in the absence of other contributing factors, the resistance factor (RF) will always be greater than or equal to the relative viscosity (Equation (3)). The ratio of RF to relative viscosity as a function of injected polymer viscosity is shown in Figure 9.

For low polymer concentrations, *RF*/*µ_rel_* values are much greater than 1. The resulting flow resistance is 2–5 times greater than that expected from bulk viscosity. The relatively high pressure generated by polymer solutions of low concentrations partly explains why the oil recovery is similar to that of solutions with higher concentrations of polymer. Another factor is the rheology of the polymer. In the classical work of Chauveteau [39], he showed that HPAM solutions exhibited exponential and abrupt increases in viscosity when meeting a certain criterion, which was a combination of polymer molecular weight, concentration, flow velocity, pore throat size, and aspect ratio. In a rock with a wide distribution of pore sizes, ranging several orders of magnitude, the velocity in the smaller pores may be above that of the onset of elastic turbulence, whereas that in the larger pores may be below that level. Although this may not significantly impact the overall measured pressure, it may significantly alter the local flow paths at the pore scale. For low-concentration (dilute) polymer solutions, the effect on the average pressure of the core is assumed to be larger as the difference between in situ viscosity in the small and large pores is expected to be relatively larger than that for high-concentration solutions in the semi-dilute region. This may partly explain why low-concentration solutions give a relatively high resistance pressure as shown in Figure 9. It is not possible to observe these pore-scale effects directly and this hypothesis is based on supporting data such as [38,39]. The role of rheology at an adverse mobility ratio was investigated by comparing the secondary displacement of oil by HPAM, xanthan, glycerol and water [17]. These fluids have distinctly different rheological behaviors, which had a significant impact on the oil recovery process, showing the importance of rheology in these mechanisms.

**Figure 10:**
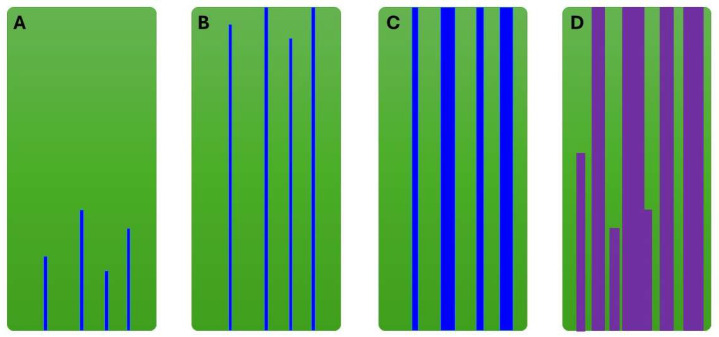
Simplified illustration of viscous finger pattern in 2D representation of core where green is oil, blue is water and purple is polymer, at four different stages: (**A**) initial finger formation, (**B**) finger propagation and WBT, (**C**) finger widening at end of water flood, and (**D**) water fingers widening into channels by polymer flow.

The discussion so far does not consider that the oil displacement occurs at an adverse mobility ratio. In our experiments, the oil viscosity is 230 cP and the oil–water viscosity ratio is 225. At this high contrast in viscosity, the water will generate thin fingers through the oil as described by [3,7,18,33] among others. Consequently, water breakthrough occurs early; in the experiments reported herein, this took place when around 0.3 pore volumes were injected. This is illustrated in a simplified schematic in Figure 10A,B. Once these fingers are established, they act as a “shortcut” for further injection. The subsequent injected water will follow the established fingers as they are the paths of least resistance. Thus, the fingers will slightly widen as illustrated in Figure 10C. When the injection is switched to polymer, it too will follow the path of least resistance—the water fingers. One can argue that this fingering pattern should lead to poor sweep and low oil mobilization. However, the state in Figure 10C being a starting point for the polymer flood explains the efficiency of the low-viscosity polymer solutions. As polymer flows through the established fingers, it adsorbs on the pore walls and blocks smaller pores, thereby strongly reducing the permeability of these fingers. Therefore, the fingers are widened into effective channels to compensate for the reduced permeability. The greater viscosity of polymer compared with water slows down the flow along the channels and exerts pressure gradients perpendicular to the flow direction, further widening the channels (Figure 10D). Note that the figure only illustrates finger widening. It is not meant to describe all of the complex characteristics of the viscous fingering phenomenon.

The oil viscosity is 230 cP, and thus there is a large mobility difference between water fingers and bypassed oil. Pressure increases as polymer enters water fingers because of the greater viscosity of the injected fluid. In addition, low polymer concentrations can be effective because of the elastic turbulence effects at the pore level, resulting in disproportionately high resistance factors (pressure) in the water channels. Consequently, the fingers widen into channels, increasing their capacity to conduct oil. Similar experiments with X-ray imaging suggest that this additional mobilization is rapid because of the crossflow of bypassed oil into the established wider channels [8].

In our experiments, low-viscosity polymer is sufficient to mobilize some of the bypassed oil. Higher-viscosity polymer does not add significantly to the recovery over low-viscosity polymer, which can be explained by the widening of fingers. Although the initial response may be higher than that for low-concentration polymer, the later response may be lower as a wider channel will lead to lower differential pressure along the channel. Low differential pressure reduces the viscous forces pushing the oil within the channel. As the channel fills with polymer, the effective permeability of the channel is further reduced. Note that the differential pressure has an inverse squared dependency on the channel radius, and thus a relatively small increase in width may lead to a relatively large decrease in pressure.

One of the main objectives of this work was to compare oil mobilization as a function of polymer viscosity in consolidated and unconsolidated rock. The difference between consolidated and unconsolidated porous media is most pronounced in imbibition processes. In the consolidated medium, the non-wetting phase is trapped by snap-off in the smaller pores. In the unconsolidated medium, fluid flow primarily occurs through the spaces between particles, allowing little trapping of the displaced phase. These differences were studied by many researchers. One of the first studies was by Naar et al. [40]. They reported that imbibition relative permeability was reduced in consolidated media compared to drainage. An opposite trend was observed in unconsolidated porous medium.

**Figure 11:**
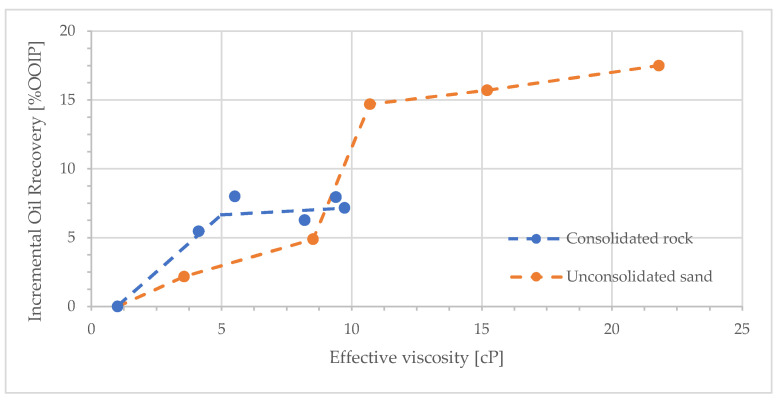
Incremental oil recovery from tertiary polymer injection as function of polymer effective viscosity for unconsolidated sand pack, as per Wang and Dong [33], and consolidated sandstone (this study). Oil viscosities are 430 and 230 cP, respectively. Dashed lines are provided to guide eye and are not fitted trends.

Here we compare our results with the sand-pack experiments reported in [33,34]. They reported additional oil production as a function of “effective viscosity”, which they defined as

(6) $$\mu_{eff}=RF\cdot \mu_{w}=\frac{\Delta P_{p}}{\Delta P_{w,prior}}\cdot \mu_{w}$$

where *µ_w_* is the brine viscosity. We calculated the “effective viscosity” in our experiments from the RF values in Table 3 and the brine viscosity in Section 2.1. A comparison of the additional oil recovery by polymer as a function of “effective viscosity” for unconsolidated sand [33] and consolidated sandstone (this study) is shown in Figure 11. Consolidated rock seems to give higher recovery at lower “effective viscosity”. This may be explained by the more open structure of the unconsolidated porous medium, which has a smaller aspect ratio, less snap-off, and lower RRF. Consequently, the in situ pressure drop is expected to be lower in unconsolidated sand packs, and the structure of viscous fingers formed during water flood would be different. These arguments may be the reason for the greater polymer concentration needed to activate extra oil production during tertiary displacement in unconsolidated sand.

The results indicate that oil mobilization by polymer injection, under adverse mobility ratio, is not directly correlated to the injected viscosity, and even low polymer concentration gives high differential pressure at adverse mobility displacements. This must be an effect of viscous fingering patterns, crossflow, and in situ porous medium rheology. Seemingly, crossflow is activated at low polymer concentrations.

Capillary end effects may influence fluid flow in porous media. They may significantly impact the interpretation of core flooding experiments and cause errors in differential pressure and oil production estimation. This is especially true in standard core analysis in which short cores are used. Therefore, the length of the cores in this study is 15 cm to minimize the impact of capillary end effects should they exist.

To investigate if capillary end effects influenced the experiments, experiment BEN07 was designed as duplicate of BEN06, with the addition of bump-rates. Three injection rates were used during water flooding and polymer flooding of BEN07, namely 0.1, 0.3 and 1 mL/min. Table 4 compares the results of the two experiments.

**Figure 12:**
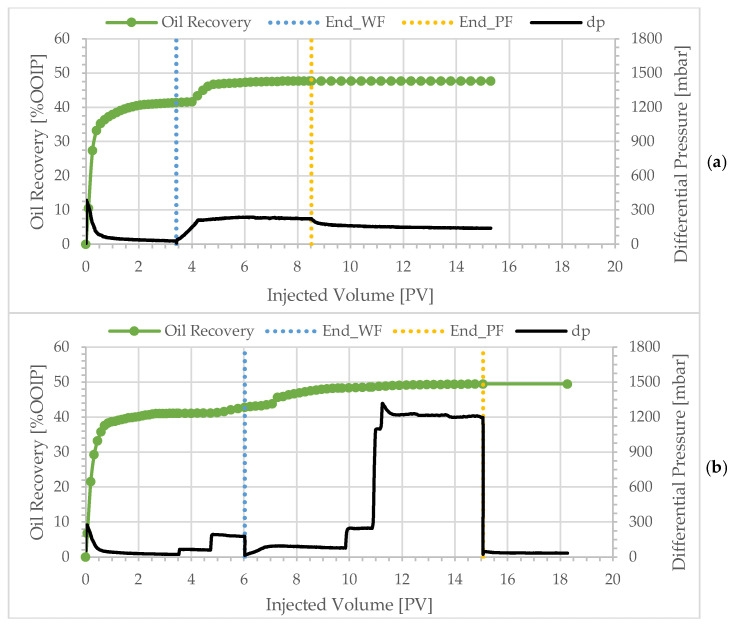
Oil recovery and differential pressure profiles at polymer viscosity of 1.8 cP: (**a**) BEN06 at constant flow rate of 0.1 mL/min; (**b**) BEN07 with bump-rates of 0.1, 0.3 and 1.0 mL/min.

Figure 12 shows the production profile and differential pressure development for the two experiments. At the end of the 0.1 mL/min water flood, oil recovery was 41.14%. Bumping the injection rate to 0.3 mL/min resulted in no significant oil production, and further bumping of the injection rate to 1.0 mL/min led to 1.57% extra oil production. Switching the injection fluid to the polymer solution at an injection rate of 0.1 mL/min resulted in 5.53% incremental recovery. Bumping the injection rate to 0.3 and 1 mL/min led to merely 0.25% and 0.9% extra oil production despite the high differential pressure (>1200 mbar). The stabilized differential pressure at the end of the 1 mL/min water flood was around 175 mbar, which was twice greater than the peak value of the differential pressure during the following 0.1 mL/min polymer flood. Moreover, the oil recovery during the 0.1 mL/min water flood—before the bump-rates—and the total oil recovery of the two experiments were in good agreement. Therefore, it is reasonable to conclude that capillary end effect does not have a significant impact on oil recovery results in these experiments.

### 3.2. Effect of Wettability Change

To investigate the effect of the wetting state of the core material on the performance of polymer flooding, experiment BEN10 was performed with the mineral oil Primol instead of crude oil. Primol was used because of its non-wetting nature, and its viscosity at ambient conditions was close to that of the crude oil. The injected polymer solution viscosity was 4.1 cP; thus, BEN10 was compared with BEN04, as presented in Table 5.

**Figure 13:**
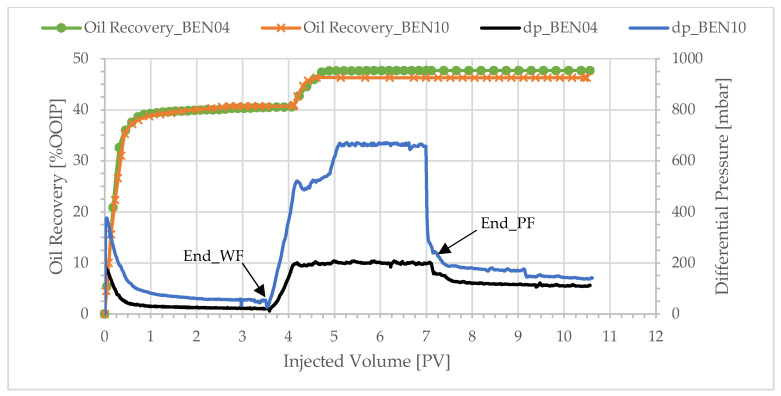
Oil recovery and differential pressure profiles of intermediate-wet core BEN04 and water-wet core BEN10 at polymer viscosity of 4.1 cP.

The water-wet state is characterized by little or no oil production after WBT in water injection core floods [9,41]. It is the result of the expansion of water films which leads to the snap-off of oil ganglia’s and to a stop in oil production after WBT at a stable mobility ratio [42]. On the contrary, at an adverse mobility ratio, significant amounts of oil may be produced after WBT. The displacement is dominated by capillary pressure [43]: the water film expansion is reduced as a result of viscosity ratio and thus little snap-off will occur. Oil production after WBT is observed in the water-wet polymer flood of BEN10 (Figure 13), indicating that unstable displacement with a strong degree of water fingering is occurring during displacement.

Intermediate-wet water flooding experiments at unit mobility ratio show substantial oil production after WBT, but at adverse mobility the displacement is significantly different [35,44,45]. As water is injected into viscous oil, water will create fingers through the oil phase and viscous fingering/crossflow will be the main displacement mechanisms.

We interpret Figure 13 under these constraints, which shows the production profiles and differential pressure development for the aged and water-wet experiments. The oil recovery from water flooding of the two experiments looks similar, but subtlety is seen at closer analysis. The two-phase production of the water-wet core is only seen in adverse mobility displacement, and the differential pressure is high, especially at the start of the water flood. The differential pressure during the polymer flood is high enough to overcome the capillary-smeared front of displacement generated during the water flood. The difference in pressure is much greater than that which can be explained by differences in core permeability (Table 1). The high differential pressure achieved by polymer injection drives an oil bank but also accelerates the waterfront.

The intermediate-wet case creates water fingers that progress rather fast through the porous media. Later polymer injection mobilizes more oil and at a lower differential pressure. This is consistent with in situ X-ray saturation measurement, in which viscous fingers dominate and the crossflow of oil into water fingers leads to mobilization at a lower differential pressure [8].

### 3.3. Effect of Injection Stage—Secondary and Tertiary Injection

**Figure 14:**
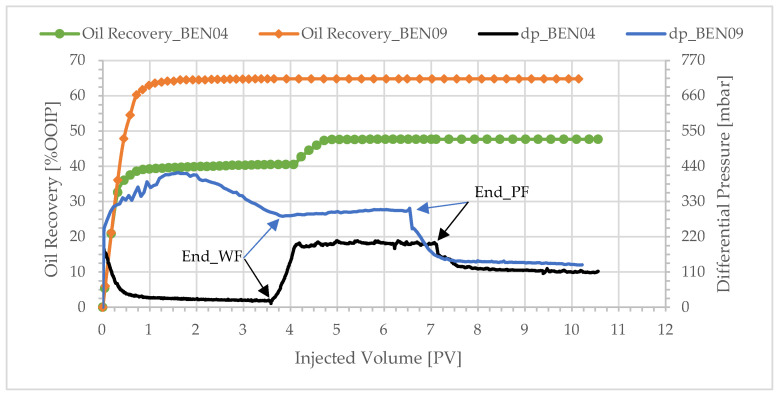
Oil recovery and differential pressure profiles of secondary polymer (BEN09) and tertiary polymer (BEN04), polymer viscosity: 4.1 cP.

To investigate the performance of polymer flooding in the secondary mode, experiment BEN09 was conducted, wherein a polymer solution was injected at an irreducible water saturation. The viscosity of the polymer solution was 4.1 cP. The experiment was compared with BEN04 in which the same polymer solution was injected in tertiary mode. Table 6 summarizes the results of the two experiments and Figure 14 shows the production profiles and differential pressures.

The total oil recovery from the secondary injection experiment was 17%OOIP greater than the tertiary injection. Overall, 97% of the recovery occurred during the first pore volume injected. The differential pressure during polymer injection was significantly higher for the secondary injection, and it stabilized slower, indicating a more chaotic displacement pattern. While the injectant breakthrough appears to be similar for both floods (at around 0.3 PV), the front does not break through before the injection of ~0.7 PV for the secondary polymer front when most of the oil has been displaced. Greater cumulative oil recovery in secondary-mode displacement was reported in other experimental studies [17,19,27,35,46] and in field tests [47]. The mechanism was discussed in [48]. X-ray CT imaging showed that, even at a 3 cP polymer solution, the viscous fingers that formed during secondary-mode polymer flooding had double the average diameter compared with water flooding [35]. Therefore, the results can be interpreted as local sweep improvement due to the complex rheology of polymer solutions in porous media. According to [35], secondary polymer injection achieved greater recovery than tertiary polymer injection; the reasons were not completely clear but were probably linked to the high water-cut seen in tertiary polymer injection. Further investigations into these mechanisms are warranted.

## 4. Conclusions

In summary, core flooding experiments were performed to investigate polymer efficiency in mobilizing viscous oil at an adverse mobility ratio. Crude oil viscosity was 230 cP, and the experiments were performed at ambient conditions on aged Bentheimer cores with similar petrophysical properties. The effect of polymer concentration, the secondary and tertiary injection of polymer, and changes in wettability were investigated.

The water floods were, in all cases, unstable and were dominated by viscous fingering. This was observed through early water breakthrough and subsequent oil production at high water-cut, leading to low recovery efficiency. Polymer injection led to an accelerated oil production, resulting in more energy-efficient displacement. Although the displacement was unstable with mobility ratio far above unity, even low concentrations of polymer lead to increased and efficient recovery. By analogy to other experiments, this is likely due to crossflow of oil into established water channels. A relatively small increase in injected fluid viscosity is sufficient to activate the crossflow. Low concentrations of polymer showed disproportionately high differential pressure relative to the injected viscosity. This could be explained by the crossflow mechanism which appeared to be enhanced by the polymer rheology exhibiting elastic turbulent properties. A further increase in polymer viscosity did not increase the recovery, suggesting that for heavy oil, lower polymer concentrations may be sufficient to increase recovery efficiency.

Increasing recovery efficiency has large benefits in field applications as it reduces the energy input in terms of water injection, lowers energy consumption in terms of lower water handling, and shortens the lifetime of the operations. This leads to a reduced carbon footprint while improving economic performance. Understanding the mechanisms of displacement at adverse mobility ratios allows for the better design of field development. These effects should be considered in addition to the traditional macroscopic improved sweep effects when estimating oil recovery from tertiary polymer injection. Because viscous fingers and crossflow are not included in conventional simulations of polymer flooding, it seems that polymer has overlooked potential that is not caught in standard modeling approaches of EOR potential.

## Figures and Tables

**Table 1:** Physical properties of the Bentheimer core material.

Parameter	BEN03	BEN04	BEN05	BEN06	BEN07	BEN08	BEN09	BEN10
length [cm]	15.10	14.93	14.30	15.10	14.62	14.91	14.83	15.14
diameter [cm]	3.80	3.73	3.73	3.78	3.80	3.72	3.79	3.80
PV [mL]	40.75	36.78	35.03	39.77	38.41	36.51	37.54	37.75
porosity [%]	23.9	22.6	22.4	23.6	23.2	22.5	22.5	22.0
$S_{wirr}$ [Fraction]	0.11	0.12	0.11	0.11	0.12	0.14	0.12	0.11
$k_{o}(S_{wirr}$) [mD]	1541	2494	2277	1446	1891	2351	1690	1615
$k_{o}(S_{wirr}$) ^1^ [mD]	1145	2496	2267	1225	1594	2215	1619	—

^1^ After aging.

**Table 2:** Summary of core flooding experiments injected with different polymer concentration.

Core ID	Injected Polymer		$S_{wirr}$ [fraction]	Recovery Factor [%OOIP]		
	Concentration [ppm]	Viscosity * [cP]		Water Flood	Polymer Flood	Final
BEN05	600	10.0	0.11	41.2	8.0	49.2
BEN04	250	4.10	0.12	40.5	7.2	47.7
BEN03	140	2.53	0.11	38.0	8.1	46.5
BEN06	70	1.80	0.11	41.4	7.1	48.5
BEN08	40	1.35	0.14	40.4	5.5	45.9

* at shear rate 10 s^−1^.

**Table 3:** Polymer bulk and in situ rheological properties.

Core ID	Conc. [ppm]	µ_rel_ (p/w) [cP/cP]	ΔP_p_ [mbar]	ΔP_w,prior_ [mbar]	RF [mbar/mbar]	RF/µ_rel_ [-]	Apparent Viscosity [cP]
BEN05	600	9.8	230	25	9.2	0.9	9.4
BEN04	250	4.0	199	21	9.5	2.4	9.7
BEN03	140	2.4	130	24	5.4	2.2	5.5
BEN06	70	1.8	225	28	8.0	4.6	8.2
BEN08	40	1.3	72	17	4.1	3.1	4.2

**Table 4:** Comparison of capillary end effect experiments.

Core ID	Injected Viscosity [cP]	$S_{wirr}$ [Fraction]	Recovery Factor [%OOIP]		
			Water Flood	Polymer Flood	Final
BEN06	1.80	0.11	41.4	7.1	48.5
BEN07 *	1.80	0.12	42.8	6.7	49.5

* bump-rates.

**Table 5:** Comparison of aged and water-wet experiments.

Core ID	Injected Viscosity [cP]	$S_{wirr}$ [Fraction]	Recovery Factor [%OOIP]		
			Water Flood	Polymer Flood	Final
BEN04 aged	4.10	0.12	40.5	7.2	47.7
BEN10 ww	4.10	0.11	40.7	5.6	46.3

**Table 6:** Comparison of secondary and tertiary polymer injection experiments.

Core ID	Injected Viscosity [cP]	$S_{wirr}$ [Fraction]	Recovery Factor [%OOIP]		
			Water Flood	Polymer Flood	Final
BEN04 (ter.)	4.10	0.12	40.5	7.2	47.7
BEN09 (sec.)	4.10	0.12	-	64.8	64.8

## Data Availability

The data presented in this study are available on request from the corresponding author due to legal reasons.

## Abbreviations

The following abbreviations are used in this manuscript:

CBM	Capillary Bundle Model
EOR	Enhanced Oil Recovery
PV	Pore Volume
RF	Resistance Factor
RRF	Residual Resistance Factor
STO	Stock Tank Oil
OOIP	Original Oil in Place
WBT	Water Breakthrough
